# Correction: Evidence for and evaluation of fluorine–tellurium chalcogen bonding

**DOI:** 10.1039/d3sc90205f

**Published:** 2023-10-18

**Authors:** Robin Weiss, Emmanuel Aubert, Loic Groslambert, Patrick Pale, Victor Mamane

**Affiliations:** a LASYROC, UMR 7177, University of Strasbourg 1 Rue Blaise Pascal 67000 Strasbourg France ppale@unistra.fr vmamane@unistra.fr; b Université de Lorraine, CNRS, CRM2 F-54000 Nancy France

## Abstract

Correction for ‘Evidence for and evaluation of fluorine–tellurium chalcogen bonding’ by Robin Weiss *et al.*, *Chem. Sci.*, 2023, **14**, 7221–7229, https://doi.org/10.1039/D3SC00849E.

The authors regret that eqn (4) was written in an incomplete form in the originally published version of the article. The correct eqn (4) is shown below.4
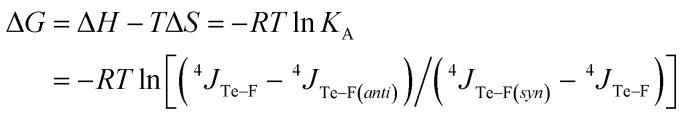


The Royal Society of Chemistry apologises for these errors and any consequent inconvenience to authors and readers.

## Supplementary Material

